# Efficacy of modified Banxia Xiexin decoction in the management of *Wei-Pi* syndrome (postprandial distress syndrome): study protocol for a randomized, waitlist-controlled trial

**DOI:** 10.1186/s13063-021-05078-y

**Published:** 2021-02-12

**Authors:** Sai Ho Sin, Jing Wu, Yuchen Kang, Kar Hung Kevin Yip, Ngo Suet Kong, Hei Wan, Bacon Fung Leung Ng, Haiyong Chen

**Affiliations:** 1 The Hong Kong Buddhist Association - The University of Hong Kong Chinese Medicine Clinic cum Training and Research Centre (Wong Tai Sin District), Hong Kong, China; 2 Department of Chinese Medicine, Hospital Authority, Hong Kong, China; 3 School of Chinese Medicine, The University of Hong Kong, 10 Sassoon Road, Pokfulam, Hong Kong

**Keywords:** Banxia Xiexin decoction, Chinese medicine, *Wei-Pi* syndrome, Postprandial distress syndrome, Functional dyspepsia, Quality of life, Randomized controlled trial, Study protocol

## Abstract

**Background:**

Postprandial distress syndrome manifests as a feeling of fullness and early satiation that can significantly reduce the quality of life of the patients. In Chinese medicine (CM), the syndrome is traditionally regarded as the *Wei-Pi* syndrome, and Banxia Xiexin decoction (BXD) has been used in the empirical treatment of the same for a long time. The current study aims to evaluate the efficacy of modified BXD in the management of *Wei-Pi* syndrome.

**Methods/design:**

A randomized, waitlist-controlled trial will be conducted. A total of 84 patients with *Wei-Pi* syndrome will be randomized into the BXD or waitlist control group in a ratio of 1:1. The patients in the BXD group will receive the semi-individualized BXD on the basis of the syndrome differentiation in CM, for a duration of 3 weeks and will be under follow-up for further 3 weeks after the completion of therapy. Conversely, the patients in the waitlist control group will undergo the same intervention and follow-up after a 3-week waiting period. In the current study, the primary outcome will be the variation in the scores pertaining to the global scale of the Quality of Life Questionnaire for Functional Digestive Disorders after 3 weeks. The secondary outcomes include the variations in the scores pertaining to the Hospital Anxiety and Depression Scale and the EuroQoL 5-dimension 5-level Questionnaire and the results of the liver and kidney function tests.

**Discussion:**

This trial will assess the efficacy of modified BXD in improving the clinical symptoms and quality of life of the patients suffering from *Wei-Pi* syndrome.

**Trial registration:**

ClinicalTrials.govNCT04398888. Registered on May 21, 2020

**Supplementary Information:**

The online version contains supplementary material available at 10.1186/s13063-021-05078-y.

## Background

Functional dyspepsia (FD) is a chronic disorder of the upper digestive tract that manifests as pain or discomfort in the upper abdomen, or a feeling of bloating, belching, fullness, and heartburn [1]. The estimated incidence of FD in the Western countries is 10 to 40% and 5 to 30% in Asia [2]. In Hong Kong, 14.6 to 18.4% of the population is affected by dyspepsia [3, 4]. The disorder imposes a significant social and economic burden on a global scale. For instance, the annual economic burden amounts to £1 billion in the UK and $18.4 billion in the USA [5, 6].

In accordance with the Rome IV criteria, FD has two subgroups: (1) epigastric pain syndrome—the patients predominantly suffer from epigastric pain or burning; (2) postprandial distress syndrome (PDS)—the patients experience a feeling of fullness and early satiation [1]. *Wei-Pi* is a term associated with gastrointestinal disorders, which is documented in the ancient Chinese medical classic, the *Huangdi’s Internal Classic* [7]. The patients with the disorder display diminution or loss of appetite, epigastric distention, and feeling of fullness. The ingestion of food aggravates the aforementioned symptoms, whereas belching relieves the same [8, 9]. As per the consensus of the China Association of Chinese Medicine (branch of Gastrointestinal Diseases), the *Wei-Pi* syndrome in Chinese medicine (CM) is equivalent to the PDS [8, 9].

A unique feature of CM is the treatment based on syndrome differentiation, wherein the patient receives an individualized herbal prescription, in line with the body constitution of the patient and the pathological factors associated with the symptoms [10, 11]. The common pathological factors that contribute to the *Wei-Pi* syndrome include exterior pathogen invasion, food stagnation, phlegm stagnation, mental dissatisfaction, and spleen-stomach vacuity [12]. *Wei-Pi* syndrome is differentiated into five patterns in Chinese medicine (CM): (1) spleen deficiency and qi stagnation, (2) liver qi invading the stomach, (3) spleen-stomach dampness-heat, (4) spleen-stomach deficiency cold, and (5) cold-heat complex pattern [8, 9]. Banxia Xiexin decoction (BXD), first documented in the CM classic, the *Treatise on Cold Damage and Miscellaneous Diseases*, is a classic formulation for the treatment of *Wei-Pi* syndrome with the cold-heat complex pattern [8, 9]. BXD is used to direct the qi downwards, in order to relieve the hiccup, dissipate the fullness and satiation, and harmonize the cold-heat in the patient’s stomach. As the clinical manifestation of symptoms may vary from patient to patient, general clinical practice adopts an approach that involves the addition of specific herbs to the BXD, in accordance with the respective syndrome differentiation (termed modified BXD). Recent clinical studies have reported that BXD is effective in the treatment of *Wei-Pi* syndrome and improves the symptoms of FD by regaining the gastric emptying rate, reducing the mucosal damage, improving the gastrointestinal function, and inducing anti-inflammatory action against *Helicobacter (H.) pylori* [13–15]. However, evidence regarding the effect of BXD on FD remains inconclusive, on account of the methodological flaws in the aforementioned studies.

The present study aims to evaluate the efficacy of modified BXD in the management of *Wei-Pi* syndrome through a randomized, waitlist-controlled trial. A semi-standardized treatment protocol based on syndrome differentiation will be adopted, in order to fulfill the CM theory on diagnosis and treatment, and to ensure scientific rigor of the trial design. A waitlist control group will be used, owing to the unavailability of an inert placebo for BXD.

## Methods/design

### Study design

This is a randomized, waitlist-controlled trial to evaluate the efficacy of semi-standardized BXD in patients with *Wei-Pei* syndrome. The study protocol follows the Declaration of Helsinki and the guidelines of the Standard Protocol Items: Recommendations for Interventional Trials (SPIRIT) and has been approved by the Institutional Review Board of the University of Hong Kong/Hospital Authority Hong Kong West Cluster (HKU/HA HKW IRB, UW 20-216) in May 2020.

### Patient recruitment

**Fig. 1:**
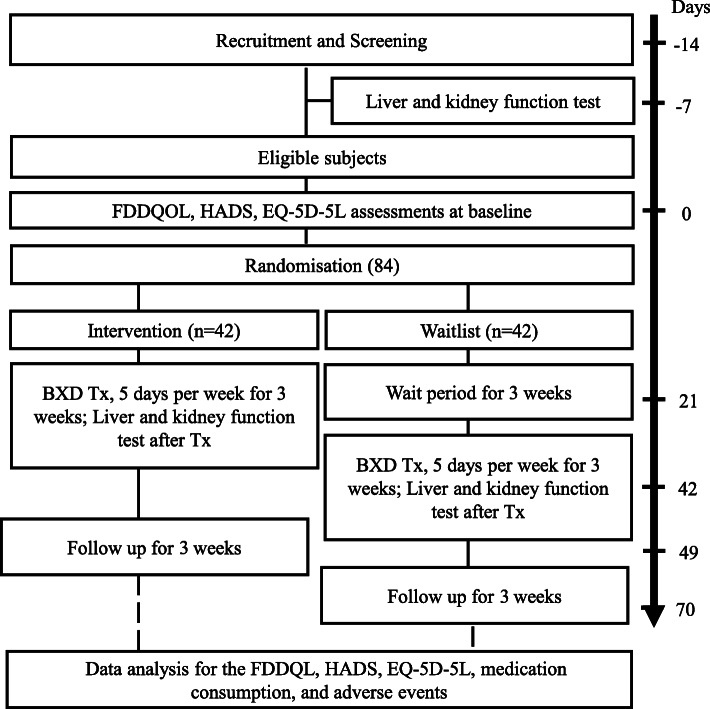
Flow diagram of patient recruitment

This trial will be conducted in the Hong Kong Buddhist Association-The University of Hong Kong Clinical Centre for Teaching and Research in Chinese Medicine (HKBA-HKU CMCTR). The subjects will be recruited using posters, webpage, Facebook page, and magazines pertaining to the Hong Kong Buddhist Association and through public dialogs and interviews in the local media. The lead investigator (SHS) will be responsible for the identification of potential recruits and obtaining informed consent. A flowchart of the study is shown in Fig. 1. The proposed project schedule is shown in Tables 1 and 2.

**Table 1:** Assessment timetable for the treatment group

	Telephone screening	Screening	Baseline (Wk 0)	Intervention			Follow-up	
				Wk 1–Wk 3			Wk 4	Wk 6
Visit number	0	1	2	3 (tel)	4 (tel)	5	6 (tel)	7
Day	− 28 to − 14	− 13 to − 7	0	7 ± 2	14 ± 2	21 ± 2	28 ± 2	42 ± 3
Screening information	✓	✓						
Informed consent		✓						
Review In/Ex criteria	✓	✓	✓	✓	✓	✓	✓	✓
Medical history	✓		✓					
Demographics			✓					
Blood test		✓					✓	
Primary outcome								
*FDDQL*			✓			✓		✓
Secondary outcomes								
*HADS*			✓			✓		✓
*EQ-5D-5L*			✓	✓	✓	✓	✓	✓
*Concomitant medications*	✓	✓	✓	✓	✓	✓	✓	✓
*Safety and tolerability*				✓	✓	✓		

*EQ-5D-5L* EuroQoL 5-dimension 5-level, *FDDQL* Quality of Life Questionnaire for Functional Digestive Disorders, *HADS* Hospital Anxiety and Depression Scale (HADS), *In/Ex* inclusion and exclusion, *Tel* telephone interview, *Wk* week

**Table 2:** Assessment timetable for the waitlist group

	Telephone screening	Screening	Baseline (Wk0)	Waiting period			Intervention			Follow-up	
				Wk1	Wk2	Wk3	Wk4–Wk6			Wk 7	Wk 9
Visit number	0	1	2	3 (tel)	4 (tel)	5	6 (tel)	7 (tel)	8	9 (tel)	10
Day	− 28 to − 15	− 13 to − 7	0	7 ± 2	14 ± 2	21 ± 2	28 ± 2	35 ± 2	42 ± 2	49 ± 2	63 ± 3
Screening information	✓	✓									
Informed consent		✓									
Review In/Ex criteria	✓	✓	✓	✓	✓	✓	✓	✓	✓	✓	✓
Medical history	✓		✓								
Demographics			✓								
Blood test		✓								✓	
Primary outcome											
*FDDQL*			✓			✓			✓		✓
Secondary outcomes											
*HADS*			✓			✓			✓		✓
*EQ-5D-5L*			✓	✓	✓	✓	✓	✓	✓	✓	✓
*Concomitant medications*	✓	✓	✓	✓	✓	✓	✓	✓	✓	✓	✓
*Safety and tolerability*							✓	✓	✓		

*EQ-5D-5L* EuroQoL 5-dimension 5-level, *FDDQL* Quality of Life Questionnaire for Functional Digestive Disorders, *HADS* Hospital Anxiety and Depression Scale (HADS), *In/Ex* inclusion and exclusion, *Tel* telephone interview, *Wk* week

### Inclusion criteria

The patients have to meet the following criteria:

1. Permanent residents of Hong Kong with ages ranging from 18 to 60 years.
2. Diagnosed with *Wei-Pi* syndrome with one or both of the following symptoms: (i) bothersome postprandial fullness, which occurs after the consumption of regular-sized meals with a minimum frequency of 3 days per week; (ii) bothersome early satiation that prevents the completion of a regular-sized meal, which occurs at a frequency of at least 3 days per week for the past 3 months; the time of onset of symptoms should be at least 6 months prior to the diagnosis [1, 9].

### Exclusion criteria

The patients will be excluded on the basis of the following criteria:

1. Pregnancy or patients planning conception
2. Abnormal liver function; patients with more than two times the upper limit of the normal range of aspartate transaminase (AST) and alanine aminotransferase levels (ALT) [16]
3. Abnormal kidney function with an estimated creatinine clearance below 80 mL/min [17]
4. History of diagnosis of gastric structural abnormalities by means of endoscopy or history of gastrectomy
5. Glucose-6-phosphate dehydrogenase deficiency
6. Alcohol or drug abuse
7. Consumption of Chinese medicine within 1 month prior to the commencement of the study
8. Unable to read/understand and sign the informed consent document

### Randomization

In the present study, 84 patients with *Wei-Pi* syndrome will be randomized into the BXD or waitlist control group in a 1:1 ratio, after obtaining informed consent. The block randomization method will be employed; random codes will be generated by means of the SPSS software and placed in opaque envelopes. The principal investigator will be responsible for the maintenance of the opaque envelopes.

### Blinding

This is an open-label study. The patients and Chinese medicine practitioners (CMPs) will be informed regarding the allocation of patients into the two groups. CMPs will perform syndrome differentiation and prescribe the modified BXD, in accordance with the patient’s pattern of differentiation. The investigators who assess the outcomes will be blinded to the allocation and will not be involved in the diagnosis and treatment. Moreover, the coded information pertaining to the patients will be used for data entry and data analysis, and the group allocations will be masked during this process.

### Intervention

#### Treatment group

Patients will be treated by CMPs with a minimum of 5 years of clinical experience. The CMPs will differentiate the syndrome and prescribe herbal medicines for the individual patient, in accordance with the semi-standardized protocol, which consists of BXD and additional herbs (Table 3).

**Table 3:** A modified BXD based on CM syndrome differentiation

Base decoction	Additional herbs when the following symptoms present
Pinellia ternate (Ban Xia) 9 g, Scutellariae Radix (Huang Qin) 9 g, *Coptis chinensis* (Huang Lian) 3 g, Zingiberis Siccatum Rhizoma (Gan Jiang) 6 g, Codonopsis Radix (Dang Shen) 9 g, Radix Glycyrrhizae (Gan Chao) 6 g, *Ziziphus zizyphus* (Da Zao) 6 g, *Cyperus rotundus* (Xiang Fu) 9 g, Hordei Fructus Germinatus (Sheng Mai Ya) 15 g	***Loss of appetite and fatigue*:** Atractylodes Macrocephala (Bai Zhu) 12 g, Codonopsis Radix (Dang Shen) 15 g (total dosage) ***Abdomen symptoms relieved by pressure and warming***: Tetradium Ruticarpum (Wu Zhuyu 5 g) ***Bitter taste, xerostomia, nausea, and yellow coating of tongue***: Gardeniae Fructus (Zhi Zi) 9 g, ArtemisiacapillarisThunb (Yin Chen) 12 g ***Gastric acid reflux***: Sepiae Endoconcha (Hai Piaoxiao) 10 g, Concha Arcae (Wa Lengzi) 15 g

The modified decoction will be prepared by means of a conventional decocting method at the HKBA-HKU CMCTR, and the auto-decocting machines will pack the decoction in bags. The patients will be instructed to consume the decoction twice per day, 5 days per week, for 3 weeks.

#### Control group

The patients in the waitlist control group will be under observation during the 3-week waiting period. Subsequently, they will undergo the same treatment and follow-up as the BXD group, in order to minimize the nocebo effect.

#### Concomitant treatment

Medications and standard care for postprandial distress syndrome will not be restricted. A patient diary will be used to document the use of medications and adverse events in the patients.

### Follow-up

All the patients will be under follow-up for a time period of 3 weeks after the completion of treatment by means of telephonic interviews or face-to-face evaluations. The schedule pertaining to the BXD and waitlist groups is shown in Tables 1 and 2, respectively. The reasons for patient dropouts or withdrawals will be recorded in the case report form.

### Outcomes

#### Primary outcome

In the present study, the primary outcome will be the variation in the scores pertaining to the global scale of the Quality of Life Questionnaire for Functional Digestive Disorders (FDDQL) after 3 weeks. FDDQL is a questionnaire with forty-three items and eight subdomains (activities, anxiety, diet, sleep, discomfort, health perceptions, coping with disease, and impact of stress), used for the evaluation of the quality of life in patients with functional digestive disorders [18]. A validated Chinese version of the FDDQL will be adopted for the study (Cantonese in Hong Kong) [19, 20].

#### Secondary outcomes

The timeline with reference to the evaluation of the secondary outcomes is shown in Tables 1 and 2.

##### Hospital Anxiety and Depression Scale

The Hospital Anxiety and Depression Scale (HADS) is a 14-item, self-administered scale used to assess the psychological distress in non-psychiatric patients [21]. As suggested by the consensus on FD, HADS is the recommended scale for the assessment of the psychological impact of *Wei-Pi* syndrome [9]. The present study will employ a validated Chinese-Cantonese version of the HADS [22].

##### EuroQoL 5-dimension 5-level

EuroQoL 5-dimension 5-level (EQ-5D-5L) is a five-item instrument used for the assessment of the generic health status, including mobility, self-care, usual activities, pain/discomfort, and anxiety/depression. The questionnaire was developed by the EuroQol Group in 2009 and is available in 130 languages (https://euroqol.org/). The EQ-5D-5L has been effectively used to measure the quality of life in patients with dyspepsia [23, 24].

##### Liver and kidney function tests

The patients will undergo liver and kidney function tests before and after the completion of therapy using modified BXD, in order to ensure patient safety and to determine the adverse effects of modified BXD. The blood AST, ALT, and creatinine levels of the patients will be evaluated using an automated chemistry analyzer (Sysmex BX-3000, Japan) in the Chung Tai X-Ray & Medical Laboratory Limited, Hong Kong.

##### Adverse events

Subjects will be asked to report any adverse events according to the monitoring form in the patient diary. The self-rated intensity of adverse events (mild, moderate, severe) and perceived causal relationship to intervention (unrelated, unlikely, possibly related, probably related, definitely related) will be recorded [25].

##### Medications

Medications that are prescribed by the doctors to relieve postprandial distress syndrome will not be restricted. The usage (dosage, frequency, and duration) of the medications will be recorded and analyzed.

### Oversight and monitoring

A trial monitoring committee (TMC) comprising experts from the Chinese Medicine Department and the Chief Pharmacist’s Office in the Hospital Authority will oversee the trial, review the safety data, and provide advice regarding protocol modification or termination of the trial. The investigators will report the progress of the trial to the TMC on a quarterly basis, including the details regarding recruitments, adverse events, issues arising from the trial, and the difficulties encountered in relation to the study.

In view of the fact that the adverse events and interim analysis will be directly reported to the TMC, the trial will not involve a data monitoring committee. Serious adverse events will be immediately reported to the TMC and the HKU/HA HKW IRB.

The HKU/HA HKW IRB will review the study protocol and protocol amendments. The investigators will regularly submit an annual report to the IRB. The project management group, consisting of the principal and lead investigators, the Chief-of-Service, and the clinical manager of the HKBA-HKU CMCTR, will convene every month, in order to review the conduct of the trial.

### Safety and risk management

Insurance will be obtained to cover any potential damage during the study. The measures that will be taken to ensure patient safety are stated as follows.

#### Quality control of herbs

All the herbs used in the study will have to meet the import standards under the Regulation of Chinese Medicine in Hong Kong (The Chinese Medicine Ordinance, Cap. 549 of the Laws of Hong Kong). The herbs will be provided by qualified CM wholesalers approved by the Hospital Authority in Hong Kong. The heavy metal levels (arsenic, mercury, and lead) in the herbs will be evaluated, in order to ensure safety [26].

#### Liver and kidney function tests

All the patients will undergo liver and kidney function tests twice (prior to and after the administration of modified BXD).

In the event of the incidental detection of abnormal liver and/or kidney functions after the consumption of Chinese medicine, the patient will be referred to a doctor for further examination, monitoring, and/or treatment.

### Ethics

The study protocol is compliant with the ICH-GCP guidelines. The collection and storage of personal data and information will follow Chapter 486 of the Personal Data (Privacy) Ordinance. All data and personal information will be stored in a locked cabinet or a password-protected computer. Documents regarding the study will be retained for 3 years after the completion of the study. Subsequently, all the documents will be destroyed.

### Statistical methods

#### Sample size

In our pilot observations, the global score of FDDQL pertaining to the patients who received BXD showed improvements with an effect size of 0.3–0.88. In the current study, the sample size was estimated with an average effect size of 0.6. The sample size required to achieve a statistical difference was estimated to be 22 patients per group (at *α* = 0.05, power = 80%, with a 95% confidence interval). Taking the dropout rate of 15% into consideration, a sample size of 84 patients (42 patients per group) is preferred. An interim analysis will be conducted to determine the adequacy of the sample size.

#### Statistical analysis

The data collection and entry into the computer will be performed by the assessors using the method of double data entry. The collected data will be sent to the statistician without identifiers. The data will be analyzed using the SPSS 24.0. The principle of intention-to-treat analysis will be used with multiple imputations. Continuous data will be analyzed by means of the *t* test or Wilcoxon rank-sum test; categorical data will be analyzed using Fisher’s exact test or Wilcoxon rank-sum test.

## Discussion

*Wei-Pi* syndrome significantly decreases the quality of life of the patients. The proposed study will assess the efficacy of a semi-individualized BXD in the management of patients with *Wei-Pi* syndrome, compared to the waitlist control group.

The current study will contribute to the methodological design of clinical trials in Chinese medicine. In routine clinical practice, CMPs perform the syndrome differentiation through a diagnostic procedure termed “four-examination” (inspection, listening and smelling, inquiry, and palpation) and tailor the Chinese herbal medicines, in accordance with the differentiated pattern. Expert consensus indicates that the *Wei-Pi* syndrome in CM is equivalent to the PDS [8, 9]. A recent study has reported that gastroenterologists exhibit a higher rate of consistency with regard to the diagnosis of the types of FD syndromes and FD-based syndrome differentiation, compared to CMPs [27]. A systematic review of the literature revealed that Chinese herbal medicine can be used as an alternative treatment for the symptoms of FD. The literature recommends that future trial designs should focus on tailoring the Chinese herbal medicines for effective management of the symptoms of FD [28]. In this study, we have designed a semi-individualized treatment protocol that combines additional herbs with the basic formula of BXD, in order to adapt to the diverse symptoms exhibited by the patients with different syndrome differentiations. This design reflects the clinical practice of Chinese herbal therapy and meets the requirements of a randomized controlled trial.

The current study has certain limitations. The *H. pylori* test and histopathology-based endoscopic examination were not included in the inclusion/exclusion criteria, on account of the limited funding. The clinical practice guidelines do not recommend the use of endoscopy to investigate the features of dyspepsia, in order to exclude upper gastrointestinal neoplasia, in patients under the age of 60 years [29]. The current study includes adult patients below the age of 60 years. The subjects involved in this study will be under follow-up for a duration of 3 weeks after the completion of the intervention. Consequently, the data collected after therapy will be comparable to the data pertaining to the subjects in the waitlist group. The short waiting period is designed to minimize the dropout of subjects in the waitlist group. The scenario warrants future studies involving a longer duration of follow-ups [28]. Chinese medicine decoctions have a unique taste and color. Accordingly, developing a physically identical placebo is a challenging endeavor [30]. The present study design involves a waitlist control group. Future trials might involve the use of capsules with herbal extraction powders as placebos, which facilitates the fabrication of an identical appearance.

In addition, the present study will assess the efficacy of modified BXD in improving the symptoms and quality of life in patients with *Wei-Pi* syndrome. The results of the current study will be reported through conferences and journals.

### Trial status

The protocol version number is 1.1, dated July 10, 2020. The patients will be recruited in August 2020. The recruitment is estimated to be completed in September 2021.

## Supplementary Information

**Additional file 1.** Participant information sheet and consent form.

## Data Availability

Access to the protocol and the dataset may be provided upon request to the authors.

## Abbreviations

ALT: Alanine aminotransferase
AST: Aspartate transaminase
BXD: Banxia Xiexin Decoction
CM: Chinese medicine
CMP: Chinese medicine practitioners
EQ-5D-5L: EuroQoL 5-dimension 5-level
FD: Functional dyspepsia
FDDQL: Quality of Life Questionnaire for Functional Digestive Disorders
HADS: Hospital Anxiety and Depression Scale
HKU/HA HKW IRB: The University of Hong Kong/Hospital Authority Hong Kong West Cluster Institutional Review Board
PDS: Postprandial distress syndrome
