# Supplementary material for: Efficacy of modified Banxia Xiexin decoction in the management of Wei-Pi syndrome (postprandial distress syndrome): study protocol for a randomized, waitlist-controlled trial
Source: Trials. 2021 Feb 12;22:135. doi: 10.1186/s13063-021-05078-y (PMC7881573; doi:10.1186/s13063-021-05078-y)
Supplement: Supplementary file 1 — Additional file 1. Participant information sheet and consent form. [file 13063_2021_5078_MOESM1_ESM.pdf]

PARTICIPANT INFORMATION SHEET

STUDY TITLE: Efficacy of a modified Banxia Xiexin Decoction (BXD) for the "*Wei Pi*" syndrome (postprandial distress syndrome): a randomized, waitlist controlled trial

Consent form version and date: 1.1 (10/07/2020)
Principal Investigator: Dr. CHEN Haiyong
Site Principal Investigator: Mr. Sai Ho Sin (RCMP)

You are invited to take part in a research study to evaluate the efficacy of a modified Banxia Xiexin Decoction (BXD) for the "*Wei Pi (胃痞)*" syndrome (postprandial distress syndrome). Before you decide, it is important for you to understand why the research is being done and what it will involve. Please read the following information carefully and discuss it with friends, relatives and doctor if you wish. Ask us if there is anything that is not clear or if you need more information.

PURPOSE OF THE STUDY

Traditional Chinese medicine has been used for *Wei Pi* syndrome (equal to postprandial distress syndrome) for a long history. The BXD is one of classic Chinese Medicine formulae for the *Wei Pi* syndrome. The purpose of this study is to examine the efficacy of a modified BXD (based on syndrome differentiation) for *Wei Pi* syndrome compared with a waitlist control.

TESTED INTERVENTION

A modified BXD will be tested. The modified BXD consists of a base BXD and several additional herbs, which are prescribed by the Chinese Medicine practitioner (CMP) to meet the patient's own symptoms.

PARTICIPANT

Hong Kong permanent resident with *Wei Pi* syndrome whose age is between 18-60 years old.

VOLUNTARY PARTICIPATION

Participation in this study is completely voluntary. If you do decide to participate you will be given this information sheet to keep and be asked to sign a consent form. If you decide to take part you are still free to withdraw at any time. This will not affect the standard of care you receive and will not affect your relationship with The Hong Kong Buddhist Association - The University of Hong Kong Chinese Medicine Clinic cum Training and Research Centre (Wong Tai Sin District).

When you complete the entire course of the study and find yourself benefiting from the treatment, you may choose to continue, but it will not be covered by the study.

STUDY DESIGN

This is a randomized, waitlist controlled trial. We aim to recruit 84 subjects. All subjects will be randomized into intervention group (n=42) and waitlist group (n=42). The study will be conducted at The Hong Kong Buddhist Association - The University of Hong Kong Chinese Medicine Clinic cum Training and Research Centre (Wong Tai Sin District).

STUDY PROCEDURE

If you decide to participate in the study, you will be invited to sign an informed consent form and receive the questionnaire screening. After the initial screening, eligible subjects will be arranged to the Chung Tai X-Ray & Medical Laboratory Limited (中大 X 光醫學化驗所有限公司) for the liver and kidney function test. Subjects satisfied the inclusion and exclusion criteria will be randomized into the intervention group or waitlist group. You will receive the liver and kidney function test before your enrollment and after the completion of the BXD intervention.

Subjects in the intervention group will receive a modified BXD for 3 weeks (oral administration twice a day, 5 days per week), and followed by a 3-week follow-up. You are required 4 visits to our clinic and 3 telephone interviews during the study. The schedule is shown in Table 1.

Table 1. Visits in the intervention group

	Screening	Baseline (Wk 0)	Intervention			Follow-up	
			Wk 1 - Wk 3			Wk 4	Wk 6
Visit number	1	2	3 (tel)	4 (tel)	5	6 (tel)	7

Subjects in the waitlist group will have a 3-week waiting period, afterward a 3-week of the modified BXD intervention, and followed by a 3-week follow-up. You are required 5 visits to our clinic and 5 telephone interviews during the study. The schedule is shown in Table 2.

Table 2. Visits in the waitlist group

	Screening	Baseline (Wk0)	Waiting period			Intervention			Follow-up	
			Wk1	Wk2	Wk3	Wk4 – Wk6			Wk 7	Wk 9
Visit number	1	2	3 (tel)	4 (tel)	5	6 (tel)	7 (tel)	8	9 (tel)	10

At the screening visit, you will be asked in detail about your age, medical history, disease duration, previous treatment, current medication. At the baseline visit, your demographic information such as date of birth, gender, height and weight, education level, marital status, annual income, occupation, cigarette, and alcohol consumption will also be collected.

During the visits, you will be required to complete several questionnaires under our assistance. It normally takes 40 minutes for a clinic visit, and 3-5 minutes for a

telephone interview.

The registered Chinese medicine practitioners (RCMPs) in our clinic will prescribe the modified BXD by taking your medical history and symptoms. The main ingredients of the modified BXD include Pinellia ternate (Ban Xia, 半夏), Scutellariae Radix (Huang Qin, 黄芩), Coptis chinensis (Huang Lian, 黄連), Zingiberis Siccatum Rhizoma (Gan Jiang, 乾薑), Codonopsis Radix (Dang Shen, 黨參), Radix Glycyrrhizae (Gan Chao, 甘草), Ziziphus zizyphus (Da Zao, 大棗), Cyperus rotundus (Xiang Fu, 香附), Hordei Fructus Germinatus (Sheng Mai Ya, 生麥芽), etc. The quality and safety of herbs meet the requirements of the Department of Health.

The modified decoction will be prepared in a conventional decocting method at the Chinese medicine pharmacy in the clinic (HKBA-HKU CMCTR), and will be packaged into bags by the auto-decocting machines. You are required to take the decoction twice per day, 5 days per week, for 3 weeks.

POTENTIAL RISKS AND EMERGENCY MEASURES

BXD is unlikely to cause serious adverse effects according to literature reviews. Chinese medicine decoction may occasionally cause vomiting, nausea, diarrhea or constipation, etc. The symptoms will be relieved when stop taking the decoction. If you have any discomforts (e.g. vomiting, nausea, diarrhea) during the study, you should inform the researcher.

Participants enrolled in the study will have the liver and kidney function test. Risks of blood draw in the test include pain, bruises, redness, swollen venous infections, and a rare risk of fainting. The tests will be performed by the nurse in the Chung Tai X-Ray & Medical Laboratory Limited.

PERSONAL DATA/ PRIVACY

Data and any other personal data collected during the course of study will be kept strictly confidential and only used for purposes related to this research. If you decide to participate in this study, your medical records may only be read and used by researcher for the purpose of data analysis. In addition, your medical and personal data may also be selected and read by regulatory authorities (The Hong Kong Buddhist Association - The University of Hong Kong Chinese Medicine Clinic cum Training and Research Centre (Wong Tai Sin District), Institutional Review Board of the University of Hong Kong /Hospital Authority Hong Kong) to make sure the study is carried out and progressing correctly and properly. Notice that any data provided by you will only be used for the purposes of this study and will be stored in our personal data system for three years and destroyed after that. Under Chapter 486 Laws of Hong Kong (Personal Data (Privacy) Ordinance), you preserve rights on the use of your personal data, such as data collection, inspection, preservation, management, control, use, transfer in or out of Hong Kong, disclosure/non-disclosure, erasure and/or in any way dealing with or disposing of any of your personal data in or for this study. . If you have any question about your legal rights, please consult the commissioner or other officers at 2827 2827 for proper monitoring or supervision of your personal data

protection so that your full awareness and understanding of the significance of compliance with the law governing privacy data is assured.

BENEFITS OF TAKING PART

We hope that the modified BXD would relieve your discomfort due to *Wei Pi* syndrome. However, since the efficacy of the modified BXD is still being evaluated, the benefit cannot be guaranteed. The results we obtain from this study will help us to provide a better treatment for patients with the disease in future.

COST AND INCENTIVES OF THE STUDY

Interventions including herbs and consultation in the study are provided free of charge. If the participant completed the intervention and follow-up, he/she will receive $100 HKD cash for their travelling costs and two free Chinese medicine consultations plus 2-day-dosage herbs in a year.

COMPENSATION AND TREATMENT FOR STUDY RELATED INJURY

If the result of your participation in this study caused any physical injury or emotional trauma, the investigator will treat you or refer you for treatment. You are not giving up any of your legal rights by signing this form.

NEW INFORMATION

During the course of study, if there is any new information becomes available and which would affect your consent of taking part, the investigator will inform you.

PARTICIPANT'S RIGHTS

Your participation in this study is voluntary. Even if you refuse to participate, you will still receive the medical services and care you deserve. You can terminate your participation during the study period and withdraw without giving reasons and will not affect the medical and nursing services you receive now or in the future. Once you have withdrawn from the study, we will continue to use the data we collected if no special requests are made. If you decide to terminate your participation in the study or have any questions, please contact Raymond Sin at 2338 3103.

If you have questions related to your rights as a research participant, please contact the Institutional Review Board of the University of Hong Kong/Hospital Authority Hong Kong West Cluster (Tel. No.: 2255 4086).

Thank you for taking part in the study.

After signing the informed consent form, you will be given a participant information sheet and a signed copy of consent form for retention.

CONSENT FORM

STUDY TITLE: Efficacy of a modified BanxiaXiexin Decoction (BXD) for the "*Wei Pi*" syndrome (postprandial distress syndrome): a randomized, waitlist controlled trial

Consent form version and date: 1.1 (10/07/2020)

Patient No:

1. I confirm that I have read and understood the information sheet for the above study and have had the opportunity to ask questions.
2. I was informed with the nature of this clinical trial, my responsibility and the possible disadvantages brought by my voluntary of taking part in this study. I have received all the written related information of this study.
3. I understand that my participation is voluntary and that I am free to withdraw at any time, without giving any reason, without my medical care or legal rights being affected.
4. I understand that sections of any of my medical notes may be read by responsible investigators or delegates from The Hong Kong Buddhist Association - The University of Hong Kong Chinese Medicine Clinic cum Training and Research Centre (Wong Tai Sin District), and the Institutional Review Board of the University of Hong Kong /Hospital Authority Hong Kong West Cluster where it is relevant to my taking part in research. I give permission for these individuals to have access to my records.
5. I agree to take part in the above study.
6. I understand that I will receive a copy of the signed and dated consent form.

Name of Participant (in Block Letter)	Signature	Date
Name of Impartial Witness (in Block Letter) (if applicable)	Signature	Date

An impartial witness's signature should be included if the participant is unable to read or write.

Name of Researcher (in Block Letter)	Signature	Date

Copies to: Subject
Researcher's File
